# The Cardiovascular Benefits of Dark Chocolate Supplementation before High-Intensity Resistance Exercise in the Early Follicular and Mid-Luteal Phases of the Menstrual Cycle

**DOI:** 10.1186/s40798-025-00850-9

**Published:** 2025-04-18

**Authors:** Chun-Wei Wang, Shih-Hua Fang, Tse-An Yu, Liang-You Chen, Chung-Kai Wang, Soun-Cheng Wang, Cheng-Shiun He

**Affiliations:** 1 Department of Sport Performance, National Taiwan University of Sport, Taichung, 404401 Taiwan; 2 Department of Athletics Sports, National Chung Cheng University, Chiayi, 621301 Taiwan; 3 Graduate Institute of Education, National Chung Cheng University, Chiayi, 621301 Taiwan

**Keywords:** Isotonic Resistance Exercise, Cocoa Flavanols, Catechins, Epicatechins

## Abstract

**Background:**

Dark chocolate, rich in flavanols, may support vascular health by reducing arterial stiffness and blood pressure across menstrual phases. This study examined the effects of 85% dark chocolate on nitric oxide (NO) levels and vascular function during high-intensity resistance exercise in healthy women across the early follicular and mid-luteal phases.

**Methods:**

Thirty-one healthy women (aged 20–30 years) with regular menstrual cycles completed a randomized, crossover study (conducted at National Chung Cheng University, Sep–Dec 2023). Participants consumed either 85% dark chocolate or milk chocolate (1 g/kg body weight) before high-intensity resistance exercise during the early follicular (days 2–5) and mid-luteal (days 18–24) phases of two menstrual cycles. Finger-toe pulse wave velocity (ftPWV), arterial stiffness, blood pressure, and plasma NO levels were measured at rest, 2 h after chocolate consumption (baseline), immediately post-exercise (T0), and at 60 (T60) and 120 (T120) minutes post-exercise.

**Results:**

Dark chocolate supplementation significantly increased NO levels and reduced systolic blood pressure (SBP), ftPWV, and arterial pressure volume index (API) (*p* < 0.05) compared to milk chocolate across both menstrual phases. During the early follicular phase, dark chocolate also attenuated exercise-induced increases in arterial stiffness and blood pressure (*p* < 0.05).

**Conclusion:**

85% dark chocolate supplementation may reduce the negative vascular effects of high-intensity resistance exercise, particularly by lowering blood pressure, arterial stiffness, and API, especially in the early follicular phase. These findings suggest that dark chocolate could be a practical, non-pharmacological intervention for improving cardiovascular health in women.

**Trial Registration:**

ClinicalTrials.gov, NCT06908941. Registered 19 March 2025 — Retrospectively registered, https://clinicaltrials.gov/study/NCT06908941.

## Key Points

1. Dark chocolate supplementation significantly improved vascular function by reducing arterial stiffness (ftPWV, API) and SBP compared to milk chocolate, irrespective of menstrual phase.

2. The vascular benefits of dark chocolate were more pronounced post-exercise during the early follicular phase, when estrogen levels were lower.

3. This study underscores the potential of dark chocolate in alleviating vascular stress induced by high-intensity resistance exercise in young women.

## Background

According to the 2020 report, the World Health Organization (WHO) indicated that cardiovascular disease (CVD) was ranked as the leading cause of death globally among the top ten causes, highlighting the significant issue of mortality due to CVD [1]. In addition to its health impact, CVD imposed a substantial economic burden due to the costs associated with medical treatment and long-term care [2]. While CVD is often perceived as a condition affecting older populations, recent studies emphasized that the risk for CVD began much earlier, with evidence suggested that cardiovascular risk factors, such as obesity, hypertension, and pregnancy-related complications, often accumulated during adolescence or early adulthood [3]. Furthermore, the incidence of acute myocardial infarction and ischemic stroke among young women was reported to be rising, driven by the cumulative effects of lifestyle-related risk factors such as physical inactivity, poor diet, and smoking [4]. Hormonal factors, such as estrogen, have also been linked to cardiovascular health. The menopausal transition, characterized by a decline in estrogen levels, was associated with impaired endothelial function, which could have begun during perimenopause and worsened with prolonged estrogen deficiency, thereby increasing cardiovascular disease risk [5]. Given these risks, research attention has been directed toward exploring non-pharmacological interventions, such as different exercise patterns, to prevent CVD and improve arterial function in healthy adults [6]. Therefore, initiating exercise training and maintaining exercise habits during the stage of healthy young women was critical for preventing the onset of cardiovascular diseases and maintaining vascular health. Both estrogen and exercise-induced changes have been suggested to play important roles in promoting vascular health and preventing cardiovascular disease in young women, highlighting the potential benefits of early interventions.

The protective effects of estrogen on vascular health were mediated through multiple mechanisms, including the enhancement of nitric oxide (NO) production, the inhibition of endothelin-1 (ET-1) activity, and the suppression of sympathetic nervous system (SNS) activity. First, estrogen enhanced NO bioavailability by activating endothelial nitric oxide synthase (eNOS), leading to improved endothelial function and vasodilation [7]. Second, estrogen reduced ET-1-mediated vasoconstriction by modulating receptor responses, diminishing ETA-mediated vasoconstriction while enhancing ETB-mediated vasodilation [8].Third, estrogen attenuated SNS activity, which contributed to reduced vascular resistance and improved hemodynamic regulation in studies involving estrogen replacement therapy [9]. Collectively, these mechanisms underscored the multifaceted role of estrogen in maintaining vascular health and mitigating cardiovascular risks. Prior research involving healthy, young, and eumenorrheic women found that higher estrogen levels were associated with reductions in the aortic reflected pressure wave and aortic augmentation index (AIx) as well as enhanced endothelial function [10]. In addition, a study focusing on women with regular menstrual cycles revealed that estrogen levels were higher during the mid luteal phase compared to the early follicular phase. In the early follicular phase, when estrogen levels were lower, pulse wave velocity (PWV) tended to be higher [11]. These findings suggested that higher estrogen levels were associated with reduced arterial stiffness. Previous studies have examined baseline (resting) blood pressure values across different menstrual phases, but the findings have been inconsistent. Some studies involving healthy adult women with regular menstrual cycles did not observe significant differences in either systolic blood pressure (SBP) or diastolic blood pressure (DBP) at rest between the follicular and luteal phases [11–13]. However, another study reported significantly higher SBP at rest in the early follicular phase compared to the late follicular and early luteal phases, along with elevated DBP at rest in the early follicular phase compared to the late follicular and early luteal phases [10]. These discrepancies highlight the need for further investigation with larger sample sizes and more comprehensive measurements. In terms of exercise and menstrual cycle effects, a study involving women with regular menstrual cycles found that high-intensity resistance exercise performed during the early follicular phase resulted in increased brachial-ankle pulse wave velocity (baPWV) compared to the mid luteal phase, where no significant changes in arterial stiffness were observed 30 and 60 min post-exercise [12]. However, blood pressure measurements taken at 30 and 60 min post-exercise showed no significant differences between the early follicular and mid luteal phases, indicating that blood pressure responses to exercise were consistent across menstrual phases [12]. These findings highlight the phase-dependent effects of estrogen on vascular responses, with higher estrogen levels in the mid luteal phase mitigating arterial stiffness following high-intensity resistance exercise. In contrast, blood pressure responses remained consistent between menstrual phases.

In past studies, researchers have observed a negative correlation between supplementing with dark chocolate and the risk of cardiovascular disease [14]. Dark chocolate is primarily derived from cocoa, which is rich in flavanols, including monomeric compounds such as catechins and epicatechins [15]. Previous research has explained the mechanisms by which flavanols improve vascular function following ingestion: (I) by stimulating the activity of nitric oxide synthase (NOS), thereby increasing the content of NO within blood vessels; (II) by inhibiting the increase in blood pressure induced by L-nitroarginine methyl ester (L-NAME), an NOS inhibitor; and (III) by suppressing the production of ET-1 [16]. In healthy adult women, a single supplementation of 85% dark chocolate or milk chocolate led to a significant reduction in blood pressure only in the dark chocolate supplementation group [17]. Similarly, acute studies conducted in healthy adults have demonstrated that dark chocolate improved vascular function by significantly reducing the AIx, indicative of decreased wave reflection, and enhancing endothelial function through increased flow-mediated dilation (FMD) [18]. Prior research investigated the acute effects of (-)-epicatechin, a primary flavanol found in cocoa, on healthy adults and reported no significant improvements in brachial artery vasodilation or exercise performance following high-intensity CrossFit^®^ resistance exercise [19]. These findings highlighted the potential vascular benefits of dark chocolate in improving endothelial function and reducing cardiovascular risk, while the lack of significant effects with isolated (-)-epicatechin supplementation following high-intensity CrossFit^®^ resistance exercise suggested that exercise modality, the complexity of cocoa polyphenols, and supplementation protocols might have influenced vascular responses. Further research is warranted to explore the combined effects of traditional resistance exercise and dark chocolate on vascular function and cardiovascular health.

Resistance training was one of the most popular forms of physical activity among young adults (aged 18–34) [20]. Long-term resistance training had been shown to improve muscular strength and endurance, enhance body composition, increase bone mineral density, and reduce cardiovascular disease risk factors in women [21]. However, acute of resistance exercise elevated arterial stiffness and blood pressure, potentially imposing additional stress on the vascular system [22]. To mitigate these acute vascular responses, it was essential to explore strategies that reduced the vascular stress induced by resistance exercise. While previous studies have separately demonstrated the vascular benefits of dark chocolate supplementation and estrogen [7–9, 14, 16, 17], the combined effects of these factors on mitigating resistance exercise-induced vascular stress remain unexplored. Furthermore, the hormonal fluctuations across the menstrual cycle provided a unique model to explore the potential protective interaction between estrogen and dark chocolate factors in response to exercise-induced stress. Therefore, this study aimed to evaluate the effects of pre-exercise supplementation with 85% dark chocolate on vascular elasticity and blood pressure responses during the early follicular and mid-luteal phases following high-intensity resistance exercise.

## Method

### Participants

This study utilized the G Power software (version 3.1.9.7, Heinrich Heine University, Düsseldorf, Germany) to calculate the required sample size. We used repeated measures analysis (ANOVA, within factors), setting a moderate effect size = 0.25, α = 0.05, power = 0.8, with 4 trials, each measured 5 times. The analysis results indicated that a minimum of 24 participants is required. Anticipating potential dropouts during the experiment, recruitment was increased to 40 participants.

This study recruited 40 healthy adult women, aged 20–30 years, from a local university campus who had a regular menstrual cycles (28 ± 7 days) in the past three months. According to Augustine et al. [13], Regecova et al. [17] and Moran et al. [23], the exclusion criteria for studies on the menstrual cycle and chocolate supplementation were adopted in this study. Participants were excluded based on the following conditions: (1) irregular menstrual cycles (menstrual cycle length < 21 or > 35 days in the past three months), (2) pregnancy or lactation in the past year, (3) history of cardiovascular or uterine surgery in the past six months, (4) use of contraceptives or other female hormone medications, (5) smoking or alcohol consumption habits, (6) hypertension (blood pressure > 140/90 mmHg), chronic diseases, heart disease, or other cardiovascular conditions, (7) allergy to cocoa products, nuts, or fruits. Before the experiment, each participant completed a self-reported questionnaire designed to assess health status, physical activity levels, and menstrual cycle regularity. The questionnaire included detailed inquiries on weekly exercise frequency and exercise duration to evaluate habitual activity levels. Participants were also asked to report whether they were members of collegiate sports teams or professional athletes, as those with high-intensity training loads were excluded to minimize confounding effects. Additionally, the questionnaire assessed medical history to confirm the absence of metabolic or cardiovascular diseases and to verify regular menstrual menstrual cycle length (28 ± 7 days) over the previous three months. In addition, participants had to read the participant information sheet carefully to understand the purpose and process of the study. This study initially recruited 40 women participants, of whom 9 were excluded due to non-adherence to protocols or ineligibility. Ultimately, 31 participants who completed the study and were included in the final analysis. National Chung Cheng University Human Research Ethics Center approved this study (ID: CCUREC111122101) and all participants provided sign the informed consent form if they voluntarily agreed to participate in the experiment. The study was conducted at National Chung Cheng University, Sep–Dec 2023.

Table 1 presents the physical and demographic characteristics of the participants, including their average age, height, weight, BMI, weekly activity levels, and one-repetition maximum (1RM) for the deadlift, bench press, and squat exercises.

### Study Design

Before the formal experiment, participants underwent four training sessions to learn correct exercise techniques (squat, bench press, and deadlift) and a 1RM test for resistance exercise to set the training intensity. One week after the training session, participants performed a 1RM test for the resistance exercises in the study, and the 1RM formula was applied to determine the 1RM values for the formal experiment [24].

**Fig. 1:**
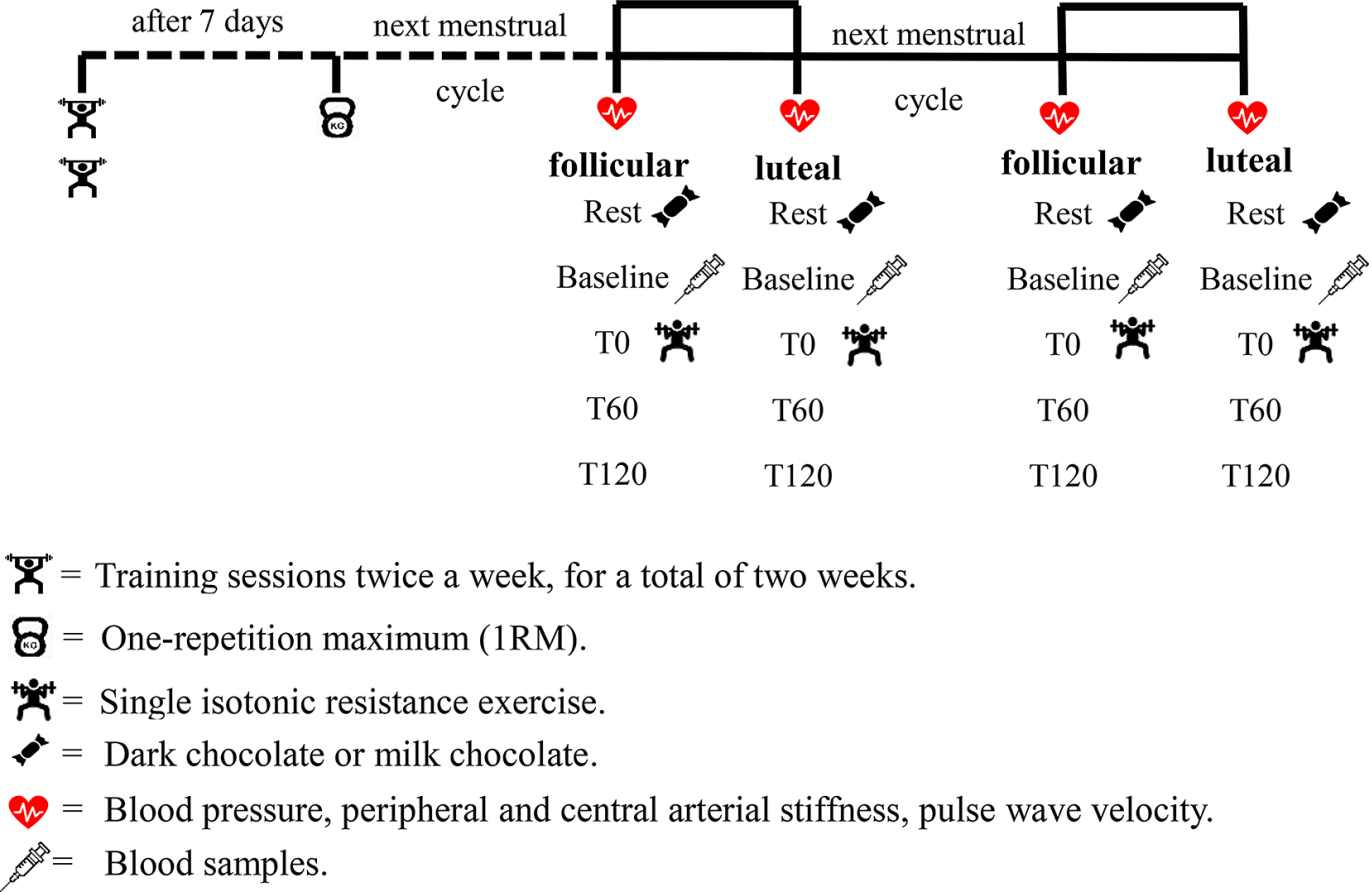
Experimental study design

Participants came to the laboratory four times for the formal experiment, conducting the experiment during the early follicular and mid luteal phases in two consecutive months. During the early follicular and mid luteal phases of the two menstrual cycles, participants underwent resistance exercise combined with dark chocolate or milk chocolate, randomly allocated. The same type of chocolate supplementation was not repeated within the same phase. Milk chocolate, which lacks the bioactive flavonoids present in dark chocolate, was selected as the control condition to the effects of flavonoid-rich dark chocolate while ensuring comparable sensory properties and caloric content [25]. Based on previous studies, significant differences in estrogen and progesterone levels during the menstrual cycle’s early follicular and mid luteal phases have been noted [11, 13]. Therefore, this study set the early follicular phase (days 2–5) and mid-luteal phase (days 18–24) for exercise in the same menstrual cycle [11]. The early follicular phase (days 2–5) was fixed for all participants, regardless of menstrual cycle length. For the mid-luteal phase, testing days were adjusted based on participants’ previous menstrual cycle lengths, which were calculated by the researchers. For example, for a 28-day menstrual cycle, the mid-luteal phase was defined as days 18–24; for a 29-day cycle, as days 19–25; and for a 27-day cycle, as days 17–23, and so forth. This approach ensured consistency across participants while limiting inclusion to regular menstrual cycles (28 ± 7 days). The first formal experiment was then conducted in the early follicular and mid luteal phases of the participants’ next menstrual cycle. On the day of the experiment, participants arrived at the laboratory between 8:00 and 10:00 a.m. Upon arrival, participants’ height and weight were measured using a calibrated stadiometer and weighing scale (Ken Zhong Weighing Instruments KC-21 A, Taiwan). Subsequently, blood pressure, peripheral and central arterial stiffness, and pulse wave velocity were measured. The experiments were conducted in a controlled environment with the laboratory temperature maintained at 22–24 °C using air conditioning. The laboratory was an independent space with minimal noise, and only the participant and research personnel were present to ensure focus and eliminate external distractions. After collecting rest values (Rest), a standard breakfast was provided consisting of russian bread (456 kcal; 10.5 g protein, 60.9 g carbohydrates) and unsweetened soy milk (40.7 kcal; 3.4 g protein, 3.5 g carbohydrates), along with supplementation of dark chocolate or milk chocolate. Two hours after supplementation, measurements of the above indicators were taken again (baseline), and blood samples were collected only at baseline. Following warm-up exercises for resistance training, isotonic resistance exercises were performed, and physiological and biochemical indexs were measured immediately after exercise (T0), at 1 h (T60), and at 120 min (T120) post-exercise, for a total of 5 measurements. The same experiment was conducted in the early follicular and mid luteal phases of the following month, following the same procedures. (Fig. 1)

### Maximum Repetition (1RM)

To avoid affecting the experimental results, the 1RM test was conducted at least 10 days apart from the formal exercise. This study referenced the method used by Hackett et al. [26] for the maximum repetition test. Before the formal test, participants underwent at least 4 training sessions. Upon arrival at the laboratory, participants began with a standard warm-up process, including dynamic training movements or static stretching. After warming up, the maximum repetition test began. The weight and number of repetitions were recorded when the participant reached muscular failure within 10 repetitions. The successful weight and number of repetitions were then used in the formula to estimate 1RM, which is 「successful weight × (1+ (0.033× successful repetitions)) 」 [27]. If the repetitions exceed 10, the load weight needs to be increased by 5–10%, and the participant should rest for 5 min before attempting again.

### Resistance Exercise

In this study, the method of isotonic resistance exercise (squat, bench press, and deadlift) was employed. These movements were selected for their effectiveness in engaging multiple muscle groups and their broad application in resistance training research [28, 29]. All three movements were conducted with participants under high safety conditions, with a certified fitness coach (Health & Exercise Association R.O.C., Taiwan) present throughout the training session. Participant responses were continuously monitored during the exercise, and if any discomfort was reported, the training was immediately halted. For the squat, during the eccentric contraction phase, the knee joint is flexed to approximately less than 90 degrees, and during the concentric contraction phase, force is applied to stand up without locking the knee joint (approximately 180 degrees), completing one repetition. For the bench press, during the eccentric contraction phase, the elbow joint is flexed to approximately less than 90 degrees, and during the concentric contraction phase, the barbell is pushed up until the arms are straight, without locking the elbow joint (approximately 180 degrees), completing one repetition. For the deadlift, the initial setup with knee joint angle of approximately 100 degrees and hip joint angle of approximately 70 degrees, stabilizing the core to keep the back straight, during the concentric contraction phase, the weight is pulled from the ground to a standing position at the hip and knee joints, without locking the joints (knee joint approximately 180 degrees, hip joint approximately 108 degrees), followed by the eccentric contraction placing the weight back on the ground, completing one repetition. The exercise training in this study began with squats, with each warm-up set starting from an empty barbell to half of the target intensity, performed for 3 sets of 5 repetitions [29], with a 3-minute rest between sets. This was followed by the formal exercise training, with the intensity set at 75% 1RM for all resistance exercises in this study, performed for 5 sets of 6 repetitions, with a 3-minute rest between sets, and a 5-minute rest between each movements. Subsequently, bench press and deadlifts were performed in the same pattern as squats. The exercise protocol was identical across all phases of the menstrual cycle to ensure consistency in training volume and intensity.

### Chocolate Supplementation

The dosage of dark chocolate supplementation was based on Regecova et al. [17], with a dosage of 1 g/kg of body weight. Participants were instructed to consume either 85% dark chocolate (DiRaja Chocolate, Pingtung, Taiwan) or milk chocolate (Hunya Foods, New Taipei City, Taiwan) (cocoa content < 25%). Both types of chocolate had similar total calorie, fat, and carbohydrate content (Table 2 for full nutritional composition). The dark and milk chocolate samples were analyzed for catechin, epicatechin, and total cocoa flavanol content at the Organic and Health Food Inspection Laboratory (OHC Lab), Chia Nan University of Pharmacy & Science, which is certified by the Taiwan of the Ministry of Health and Welfare. All chocolate was consumed alongside a standardized breakfast during each of the four formal experiments.

### Finger-Toe Pulse Wave Velocity (ftPWV)

This study used non-invasive ftPWV as an indicator of arterial stiffness. In addition, previous research has demonstrated a significant correlation between ftPWV and the gold-standard cfPWV, supporting the validity of ftPWV as a reliable measure of arterial stiffness [30]. For measurements at the Rest and Baseline time points, participants must lying flat quietly and rest for 15 min upon arrival at the laboratory to stabilize their physiological signs before beginning the ftPWV measurements. Measurements at other time points are taken while participants are resting in the laboratory. Firstly, sensors for photoplethysmography (PPG) were placed on the right index finger and right big toe of the participants. These sensors were marked with an oily pen to ensure consistent placement throughout the experiment. The signals were then amplified using a Biopec MP150 (MP150, Biopac Systems Inc., Goleta, CA, USA), with each physiological signal collected for at least 150 s. The signals were analyzed using MATLAB to calculate the time difference between the two pulse waves. The length of the participant’s body segments ( sternum to the right index finger, and sternum to the big toe of the right foot) was measured, and this value was divided by the average difference between 10 pulse waves (in the stable interval of 150 s) to calculate the ftPWV value [31, 32]. The coefficient of variation (CV) for ftPWV measurements was below 5%.

### Blood Pressure and Artery Stiffness

After completing ftPWV measurements, participants were measured for blood pressure and arterial stiffness measurements at each time point. The measured indicators include SBP, DBP, heart rate, arterial pressure volume index (API), and arterial velocity pulse index (AVI), all of which are measured using the PASESA AVE-2000 (PASESA, Tokyo, Japan) with a wrapped around the right upper arm of seated participants. MAP and PP were derived from the SBP and DBP measurements. MAP and PP were subsequently derived from the SBP and DBP measurements. MAP was calculated using the formula: MAP = DBP + [(SBP - DBP) / 3], and PP was calculated as the difference between SBP and DBP (PP = SBP - DBP). The API reflected the stiffness of peripheral arteries by assessing the pressure-volume relationship of the artery, while the AVI primarily reflected the stiffness of central arteries by analyzing the ratio of forward and reflected pressure waves [33, 34]. Each measurement was taken three times, and the average of the three readings was recorded. The CV was below 5% for SBP and DBP measurements, and below 10% for API and AVI measurement.

### Blood Analysis

To quantify the concentrations of NO and estradiol, blood samples were drawn from the antecubital vein using standard venipuncture techniques. Blood was collected into EDTA tubes, immediately centrifuged at 3,000 rpm for 10 min to separate plasma, and the plasma samples were stored at -20 °C until analysis.

Plasma concentrations of NO and estradiol were quantified using enzyme-linked immunosorbent assay (ELISA) kits. NO was assessed using the Nitrate/Nitrite Colorimetric Assay Kit (Cayman Chemical, Ann Arbor, MI, USA), and estradiol was measured using the Estradiol ELISA Kit (DRG Instruments, Marburg, Germany). All assays were performed following the manufacturers’ instructions, and each sample was analyzed in duplicate. The CV for both assays was maintained below 5%.

### Statistical Analysis

The recorded data were analyzed using the statistical software SPSS (version 23.0, IBM Inc., Armonk, NY, USA). Descriptive statistics were used to calculate the mean and standard deviation of the participants’ basic information. Normality of the data was assessed using the Shapiro-Wilk test. A two-way repeated measures analysis of variance (ANOVA) was conducted to compare the effects of different trials on the dependent variables. The trials included early follicular milk chocolate (EF-MC), early follicular dark chocolate (EF-DC), mid luteal milk chocolate (ML-MC), and mid luteal dark chocolate (ML-DC). Measurements were taken at five time points: pre-chocolate and pre-exercise (Rest), 2 h after consuming chocolate (Baseline-C), immediately after exercise (T0), 60 min after exercise (T60), and 120 min after exercise (T120). The analysis examined the trial × time interaction to determine how these factors influenced the outcomes. If a significant interaction was found, Bonferroni post hoc tests were conducted to examine the specific effects of each factor on the results. Additionally, one-way repeated measures ANOVA was performed to compare changes in blood samples, including NO and estrogen levels, across the four trials (EF-MC, EF-DC, ML-MC, ML-DC). The significance level was set at *p* < 0.05.

## Results

**Table 1:** Participant characteristics (*N* = 31)

Variable	Means ± SD	Range (Min-Max)
Age (year)	22.10 ± 1.85	20.00–27.00
Height (cm)	161.26 ± 6.97	149.00-182.00
Weight (kg)	55.47 ± 6.06	42.50–70.10
Chocolate Intake (g)	55.47 ± 6.06	42.50–70.10
BMI (kg/m2)	21.34 ± 1.99	16.81–25.22
Activity (min/week)	115.00 ± 108.74	10.00-480.00
1-RM Deadlift (kg)	72.19 ± 12.52	44.00-100.00
1-RM Bench Press (kg)	27.74 ± 6.86	17.00–47.00
1-RM Squat (kg)	54.84 ± 9.48	39.00–77.00
Menstrual Cycle (days)	31.10 ± 2.33	27.00–35.00

Note BMI: body mass index; 1RM: 1repetition maximum

**Table 2:** Nutritional composition per 100 g of milk and dark chocolate

Nutritional component	Milk chocolate	Dark chocolate
Energy (kJ)	2269.8	2618.7
Protein (g)	8.3	12.2
Total fat (g)	32.8	49.1
Saturated fat (g)	32.0	27.7
Trans fat (g)	0.0	0.0
Carbohydrates (g)	53.8	33.8
Sugars (g)	49.8	14.5
Sodium (mg)	87.5	0.0
Total cocoa flavanol (mg)	773.0	4370.0
catechin (mg)	58.3	659.5
epicatechin (mg)	75.9	376.7

**Table 3:** The effects of dark or milk chocolate supplementation with a standard breakfast on NOx (nitrate/nitrite) and estradiol levels 2 h post-ingestion during different menstrual cycles

	EF-MC	EF-DC	ML-MC	ML-DC
Estradiol (pg/ml)	66.53 ± 21.24	62.19 ± 24.06	128.79 ± 58.54^a^	127.11 ± 64.26 ^a^
Nitrite/Nitrate (µmol/l)	24.26 ± 13.84	32.14 ± 14.16^b^	25.89 ± 11.67	30.31 ± 12.64^b^

Note The value are mean ± SD; EF-MC = early follicular-milk chocolate; EF-DC = early follicular-dark chocolate; ML-MC = Mid Luteal-milk chocolate; ML-DC = Mid Luteal-dark chocolate

^a^ = Significant difference compared with EF-MC and EF-DC trials (*p* < 0.05)

^b^ = Significant difference compared with EF-MC and ML-MC trials (*p* < 0.05)

### Plasma NOx and Estrogen Responses

This study assessed the plasma levels of NOx to evaluate the vascular differences following a 2-hour supplementation with dark or milk chocolate. Additionally, estradiol levels were measured to confirm menstrual cycle phases, as presented in Table 3. In the Estradiol indicator, the ML-MC and ML-DC trials were significantly higher levels than the EF-MC and EF-DC trials (*p* < 0.05). Additionally, in the NOx indicator, the EF-DC and ML-DC trials showed significantly higher levels than the EF-MC and ML-MC trials (*p* < 0.05).

**Fig. 2:**
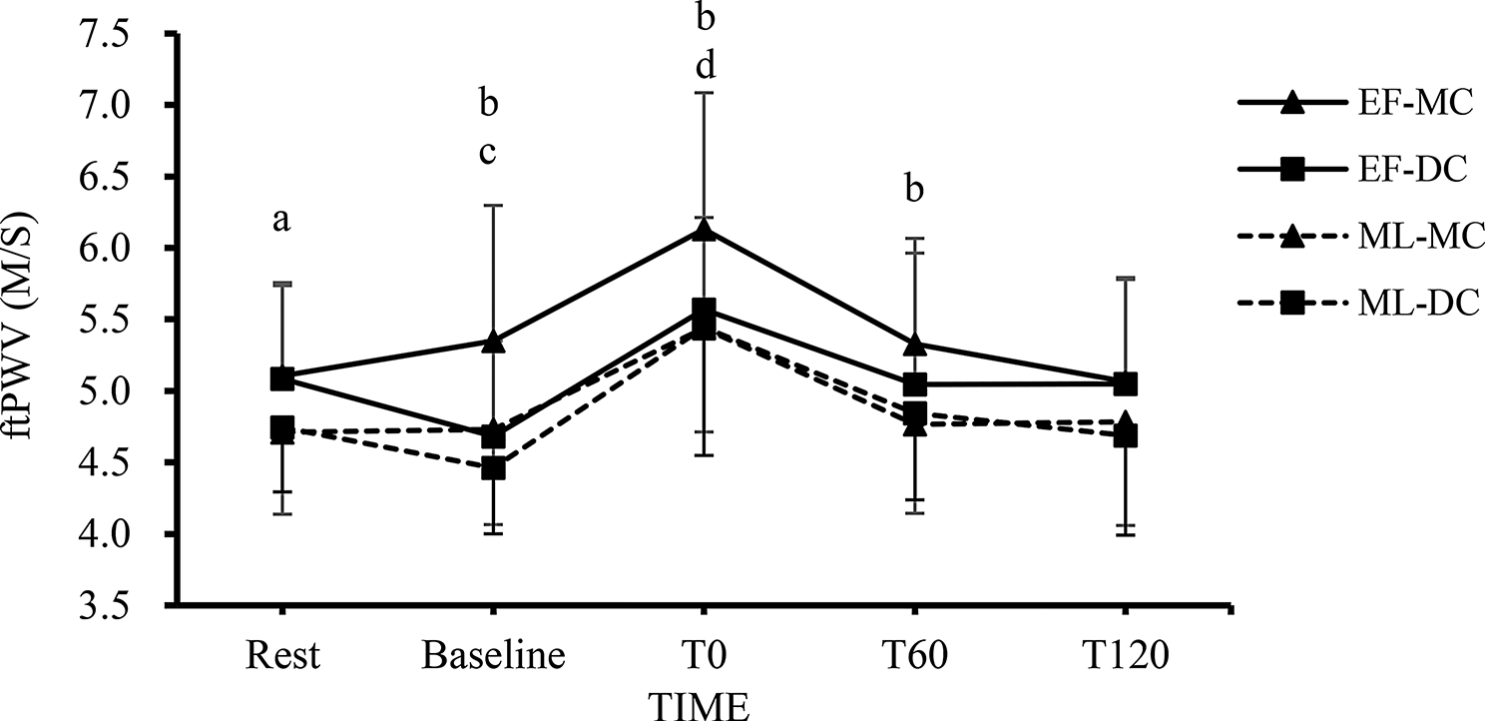
The changes in ftPWV at each time point in the EF-MC, EF-DC, ML-MC, and ML-DC trials. Note. The value are mean ± SD; EF-MC: early follicular-milk chocolate; EF-DC: early follicular-dark chocolate; ML-MC: Mid Luteal-milk chocolate; ML-DC: Mid Luteal-dark chocolate; ftPWV: finger-toe pulse wave velocity; Rest: pre-chocolate and pre-exercise; Baseline: 2 h after consuming chocolate; T0: 0 min after exercise; T60: 60 min after exercise; T120: 120 min after exercise. **a** = The EF-MC and EF-DC trials showed a significant difference compared to both the ML-MC and ML-DC trials (*p* < 0.05). **b** = The EF-MC trial showed a significant difference compared to the EF-DC, ML-MC and ML-DC trials (*p* < 0.05). **c** = Significant difference against rest in the EF-MC, EF-DC and ML-DC trials (*p* < 0.05). **d** = Significant difference against rest in the all trials (*p* < 0.05)

### FtPWV Responses

Figure 2 shows changes in ftPWV at rest, baseline, 0, 60, and 120 min after resistance exercise, supplemented with either dark chocolate or milk chocolate, across each menstrual cycle. The two-way repeated measures ANOVA revealed a significant main effect of trials on ftPWV (*F* = 15.546, *p* < 0.001, $\:{\eta\:}_{p}^{2}$ = 0.349) and a significant main effect of time (*F* = 56.023, *p* < 0.001, $\:{\eta\:}_{p}^{2}$ = 0.659). Additionally, a significant trial × time interaction effect was observed (*F* = 2.970, *p* = 0.001, $\:{\eta\:}_{p}^{2}$ = 0.093). Post hoc Bonferroni comparisons revealed that ftPWV was significantly higher in the EF-MC and EF-DC trials at rest compared with the ML-MC and ML-DC trials (*p* < 0.05). The ftPWV was significantly higher in the EF-MC trial at Baseline, T0 and T60 compared with the EF-DC, ML-MC and ML-DC trials (*p* < 0.05). In addition, significant increases in ftPWV were observed from rest to baseline in the EF-MC trial (*p* < 0.05). The ftPWV in the EF-DC and ML-DC trials showed significant decrease at baseline when compared to the rest condition (*p* < 0.05). All trials exhibited significant increases in ftPWV at T0 after resistance exercise (*p* < 0.05).

**Fig. 3:**
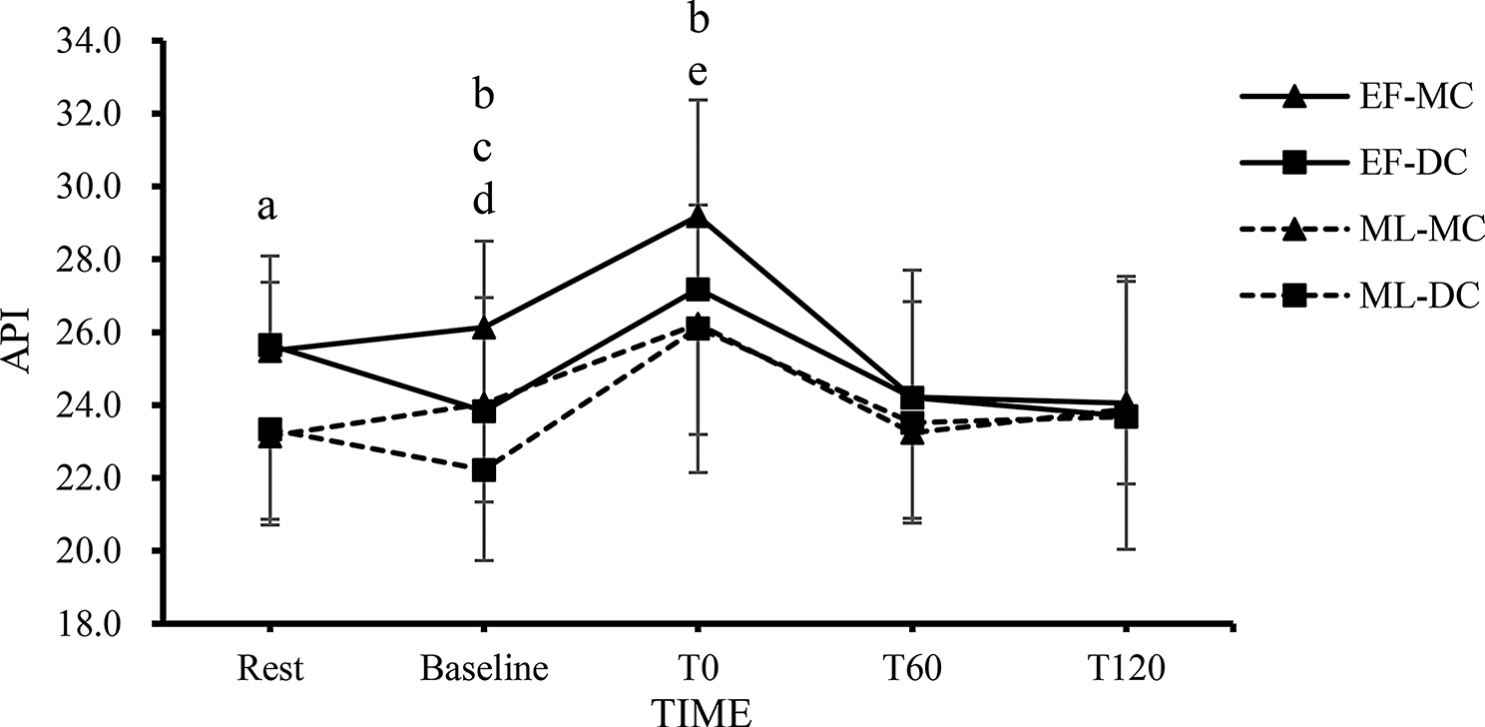
The changes in API at each time point in the EF-MC, EF-DC, ML-MC, and ML-DC trials. Note. The value are mean ± SD; EF-MC: early follicular-milk chocolate; EF-DC: early follicular-dark chocolate; ML-MC: Mid Luteal-milk chocolate; ML-DC: Mid Luteal-dark chocolate; API: arterial pressure volume index; Rest: pre-chocolate and pre-exercise; Baseline: 2 h after consuming chocolate; T0: 0 min after exercise; T60: 60 min after exercise; T120: 120 min after exercise. **a** = The EF-MC and EF-DC trials showed a significant difference compared to both the ML-MC and ML-DC trials (*p* < 0.05). **b** = The EF-MC trial showed a significant difference compared to the EF-DC, ML-MC and ML-DC trials (*p* < 0.05). **c** = The ML-MC trial showed a significant difference compared to the ML-DC trial (*p* < 0.05). **d** = Significant difference against rest in the EF-DC and ML-DC trials (*p* < 0.05). **e** = Significant difference against rest in the all trials (*p* < 0.05)

### API Responses

Figure 3 shows changes in API at rest, baseline, 0, 60, and 120 min after resistance exercise, supplemented with either dark chocolate or milk chocolate, across each menstrual cycle. The two-way repeated measures ANOVA revealed a significant main effect of trials on API (*F* = 6.389, *p* = 0.001, $\:{\eta\:}_{p}^{2}$ = 0.203) and a significant main effect of time (*F* = 34.164, *p* < 0.001, $\:{\eta\:}_{p}^{2}$ = 0.611). Additionally, a significant trial × time interaction effect was observed (*F* = 3.443, *p* < 0.001, $\:{\eta\:}_{p}^{2}$ = 0.112). Post hoc Bonferroni comparisons revealed that API was significantly higher in the EF-MC and EF-DC trials at rest compared with the ML-MC and ML-DC trials (*p* < 0.05). The API was significantly higher in the EF-MC trial at Baseline and T0 compared with the EF-DC, ML-MC and ML-DC trials (*p* < 0.05). The API was significantly higher in the ML-MC trial at Baseline compared with the ML-DC trials (*P* < 0.05). The API in the EF-DC and ML-DC trials showed significant decrease at baseline when compared to the rest condition (*p* < 0.05). All trials exhibited significant increases in API at T0 after resistance exercise (*p* < 0.05).

**Fig. 4:**
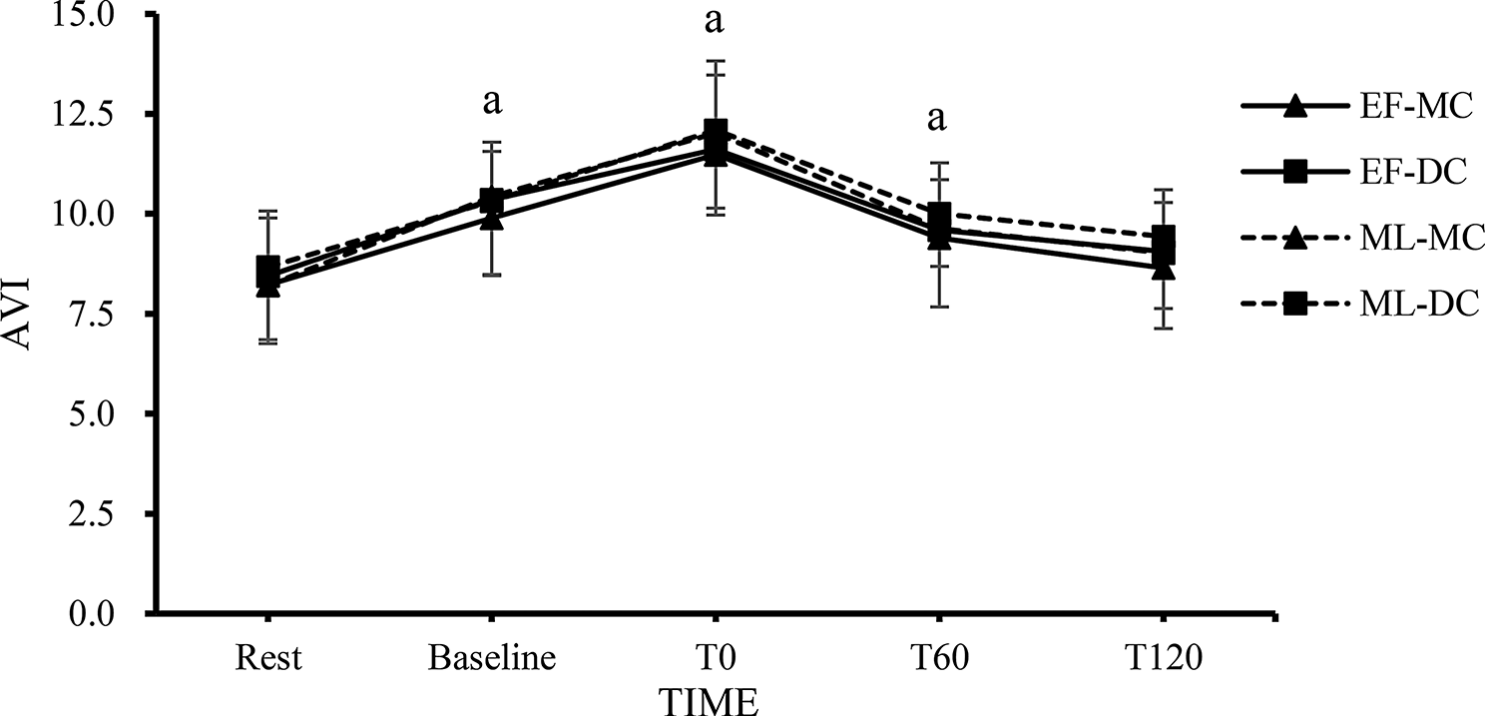
The changes in AVI at each time point in the EF-MC, EF-DC, ML-MC, and ML-DC trials. Note. The value are mean ± SD; EF-MC: early follicular-milk chocolate; EF-DC: early follicular-dark chocolate; ML-MC: Mid Luteal-milk chocolate; ML-DC: Mid Luteal-dark chocolate; AVI: arterial velocity pulse index; Rest: pre-chocolate and pre-exercise; Baseline: 2 h after consuming chocolate; T0: 0 min after exercise; T60: 60 min after exercise; T120: 120 min after exercise. a = Significant difference against rest in the all trials (*p* < 0.05)

### AVI Responses

Figure 4 shows changes in AVI at rest, baseline, 0, 60, and 120 min after resistance exercise, supplemented with either dark chocolate or milk chocolate, across each menstrual cycle. The two-way repeated measures ANOVA revealed no significant main effect of trials on AVI (*F* = 1.773, *p* = 0.159, $\:{\eta\:}_{p}^{2}$ = 0.064) and a significant main effect of time (*F* = 65.110, *p* < 0.001, $\:{\eta\:}_{p}^{2}$ = 0.715). Additionally, the trial × time interaction effect was not significant (*F* = 0.293, *p* = 0.990, $\:{\eta\:}_{p}^{2}$ = 0.011). Post hoc Bonferroni comparisons revealed that all trials exhibited significant increases in AVI at baseline, T0 and T60 after resistance exercise (*p* < 0.05).

**Fig. 5:**
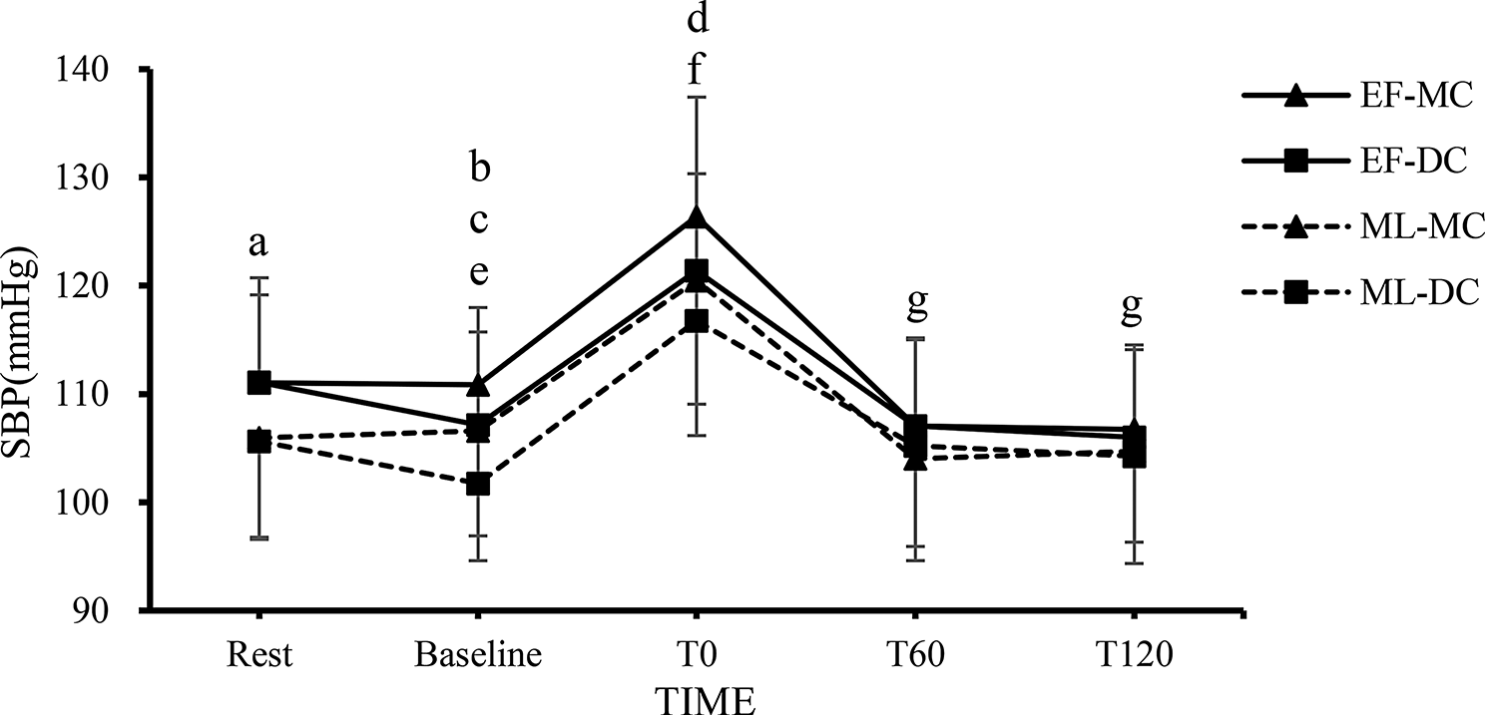
The changes in SBP at each time point in the EF-MC, EF-DC, ML-MC, and ML-DC trials. Note. The value are mean ± SD; EF-MC: early follicular-milk chocolate; EF-DC: early follicular-dark chocolate; ML-MC: Mid Luteal-milk chocolate; ML-DC: Mid Luteal-dark chocolate; SBP: systolic blood pressure; Rest: pre-chocolate and pre-exercise; Baseline: 2 h after consuming chocolate; T0: 0 min after exercise; T60: 60 min after exercise; T120: 120 min after exercise. **a** = The EF-MC and EF-DC trials showed a significant difference compared to both the ML-MC and ML-DC trials (*p* < 0.05). **b** = The EF-MC trial showed a significant difference compared to the EF-DC, ML-MC and ML-DC trials (*p* < 0.05). **c** = The ML-DC trial showed a significant difference compared to the EF-MC, EF-DC and ML-MC trials (*p* < 0.05). **d** = The EF-MC trial showed a significant difference compared to the ML-DC trial (*p* < 0.05). **e** = Significant difference against rest in the EF-DC and ML-DC trials (*p* < 0.05). **f** = Significant difference against rest in the all trials (*p* < 0.05). **g** = Significant difference against rest in the EF-MC and EF-DC trials (*p* < 0.05)

### SBP Responses

Figure 5 shows changes in SBP at rest, baseline, 0, 60, and 120 min after resistance exercise, supplemented with either dark chocolate or milk chocolate, across each menstrual cycle. The two-way repeated measures ANOVA revealed a significant main effect of trials on SBP (*F* = 9.957, *p* < 0.001, $\:{\eta\:}_{p}^{2}$ = 0.323) and a significant main effect of time (*F* = 82.613, *p* < 0.001, $\:{\eta\:}_{p}^{2}$ = 0.796). Additionally, a significant trial × time interaction effect was observed (*F* = 6.235, *p* = 0.018, $\:{\eta\:}_{p}^{2}$ = 0.081). Post hoc Bonferroni comparisons revealed that SBP was significantly higher in the EF-MC and EF-DC trials at rest compared with the ML-MC and ML-DC trials (*p* < 0.05). The SBP was significantly higher in the EF-MC trial at Baseline compared with the EF-DC, ML-MC and ML-DC trials (*p* < 0.05). The SBP was significantly higher in the ML-DC trial at Baseline compared with the EF-MC, EF-DC and ML-MC trials (*p* < 0.05). The SBP was significantly higher in the ML-MC trial at T0 compared with the ML-DC trial (*p* < 0.05). The SBP in the EF-DC and ML-DC trials significantly decreased from rest to baseline (*p* < 0.05). All trials exhibited significant increases in SBP at T0 after resistance exercise (*p* < 0.05). The SBP in the EF-MC and EF-DC trials showed significant decrease at T60 and T120 when compared to the rest condition (*p* < 0.05).

**Table 4:** The changes in blood pressure and heart rate at each time point in the EF-MC, EF-DC, ML-MC, and ML-DC trials

		Rest	Baseline	T0	T60	T120	Trial; Time; Trial×Time
DBP (mmHg)	EF-MC	67.48 ± 6.69	67.42 ± 8.00	76.26 ± 7.94^d^	67.52 ± 5.33	68.02 ± 6.65	*p* = 0.307; *p* < 0.001; *p* = 0.376
	EF-DC	66.85 ± 7.51	68.92 ± 7.27	76.85 ± 8.81^d^	70.10 ± 6.29	68.95 ± 8.29	
	ML-MC	67.94 ± 8.44	67.58 ± 7.28	75.84 ± 8.97^d^	66.21 ± 5.87	66.89 ± 7.30	
	ML-DC	67.15 ± 8.99	68.98 ± 7.29	75.66 ± 8.61^d^	68.89 ± 7.38	68.42 ± 8.51	
MAP (mmHg)	EF-MC	82.01 ± 6.04	81.90 ± 6.96	92.97 ± 7.03^cd^	80.69 ± 5.29	80.92 ± 5.82	*p* = 0.006; *p* < 0.001; *p* = 0.207
	EF-DC	81.59 ± 6.92	81.66 ± 6.63	91.70 ± 7.56^d^	82.41 ± 5.91	81.30 ± 7.31	
	ML-MC	80.61 ± 7.60	80.59 ± 6.68	90.72 ± 8.31^d^	78.81 ± 5.59	79.50 ± 6.47	
	ML-DC	79.97 ± 7.98	79.90 ± 6.36	89.36 ± 7.16^d^	81.00 ± 7.43	80.36 ± 7.83	
PP (mmHg)	EF-MC	43.56 ± 8.28^b^	43.45 ± 7.05	50.30 ± 12.37 ^cd^	39.53 ± 7.37^d^	38.71 ± 8.46^d^	*p* < 0.001; *p* < 0.001; *p* = 0.017
	EF-DC	44.21 ± 9.67^b^	38.21 ± 8.49^ad^	44.53 ± 9.79	36.94 ± 7.84^d^	37.05 ± 8.01^d^	
	ML-MC	38.02 ± 9.27	39.03 ± 10.01	44.63 ± 11.23^d^	37.81 ± 9.76	37.84 ± 8.81	
	ML-DC	38.48 ± 8.57	33.08 ± 6.67^ad^	41.10 ± 12.67	36.34 ± 6.70	35.82 ± 9.39	
HR (bpm)	EF-MC	72.65 ± 9.03	79.21 ± 8.77^d^	101.48 ± 11.68^d^	80.11 ± 8.08^d^	75.13 ± 9.28	*p* = 0.119; *p* < 0.001; *p* = 0.792
	EF-DC	72.87 ± 8.38	79.37 ± 6.99^d^	101.68 ± 14.32^d^	82.48 ± 8.35^d^	77.19 ± 8.59	
	ML-MC	75.40 ± 9.24	81.55 ± 6.82^d^	101.84 ± 16.51^d^	82.24 ± 8.49^d^	77.52 ± 7.71	
	ML-DC	74.79 ± 8.25	80.06 ± 6.53^d^	102.84 ± 13.56^d^	83.24 ± 10.73^d^	79.21 ± 8.95	

Note The value are mean ± SD; EF-MC = early follicular-milk chocolate; EF-DC = early follicular-dark chocolate; ML-MC = Mid Luteal-milk chocolate; ML-DC = Mid Luteal-dark chocolate; DBP: diastolic blood pressure; PP: pulse pressure; MAP: mean arterial pressure; HR: heart rate; Rest: pre-chocolate and pre-exercise; Baseline: 2 h after consuming chocolate; T0: 0 min after exercise; T60: 60 min after exercise; T120: 120 min after exercise

^a^ = Significant difference were evaluated within the same menstrual phase (EF-MC vs. EF-DC; ML-MC vs. ML-DC) (*p* < 0.05)

^b^ = Significant difference were evaluated between menstrual phases (EF-MC and EF-DC vs. ML-MC and ML-DC) (*p* < 0.05)

^c^ = The EF-MC trial showed a significant difference compared to the ML-DC trial (*p* < 0.05)

^d^ = Significant difference against rest within the same trial (*p* < 0.05)

### Blood Pressure and Heart Rate Responses

Table 4 shows changes in blood pressure and heart rate at rest, baseline, 0, 60, and 120 min after resistance exercise, supplemented with either dark chocolate or milk chocolate, across each menstrual cycle. The two-way repeated measures ANOVA revealed no significant main effect of trials for DBP (*F* = 1.221, *p* = 0.307, $\:{\eta\:}_{p}^{2}$ = 0.039) and HR (*F* = 2.002, *p* = 0.119, $\:{\eta\:}_{p}^{2}$ = 0.063), while significant trial effects were found for MAP (*F* = 4.384, *p* = 0.006, $\:{\eta\:}_{p}^{2}$ = 0.128) and PP (*F* = 11.629, *p* < 0.001, $\:{\eta\:}_{p}^{2}$ = 0.279). Significant main effects of time were observed for DBP (*F* = 60.756, *p* < 0.001, $\:{\eta\:}_{p}^{2}$ = 0.669), MAP (*F* = 121.057, *p* < 0.001, $\:{\eta\:}_{p}^{2}$ = 0.801), PP (*F* = 25.377, *p* < 0.001, $\:{\eta\:}_{p}^{2}$ = 0.458), and HR (*F* = 166.981, *p* < 0.001, $\:{\eta\:}_{p}^{2}$ = 0.848). No significant trial × time interaction effects were found for DBP (*F* = 1.079, *p* = 0.376, $\:{\eta\:}_{p}^{2}$ = 0.035), MAP (*F* = 1.316, *p* = 0.207, $\:{\eta\:}_{p}^{2}$ = 0.042), and HR (*F* = 0.658, *p* = 0.792, $\:{\eta\:}_{p}^{2}$ = 0.021), while a significant trial × time interaction effect was found for PP (*F* = 2.092, *p* = 0.017, $\:{\eta\:}_{p}^{2}$ = 0.065). Post hoc Bonferroni comparisons revealed that PP was significantly lower in the EF-DC trial compared to EF-MC trial, and ML-DC trial compared to ML-MC trial at Baseline (*p* < 0.05). The PP was significantly higher in the EF-MC and EF-DC trials at Rest compared with the ML-MC and ML-DC trials (*p* < 0.05). The MAP and PP were significantly higher in the EF-MC at T0 compared with the ML-DC (*p* < 0.05). For DBP and MAP, significant increases was observed from Rest to T0 in all trials (*p* < 0.05). For PP, significant decreases was observed from Rest to Baseline in EF-DC and ML-DC trials (*p* < 0.05), significant increases were observed from Rest to T0 in EF-MC and EF-DC trials (*p* < 0.05), significant decreases were observed from Rest to T60 and T120 in EF-MC and EF-DC trials (*p* < 0.05). For HR, significant increases were observed from Rest to Baseline, T0 and T60 in all trials (*p* < 0.05).

## Discussion

Our study demonstrated that dark chocolate supplementation, regardless of the menstrual phase, resulted in significantly lower ftPWV, API, SBP and PP compared to milk chocolate consumption. Additionally, NO levels were markedly higher following dark chocolate consumption, which may play a key role in preventing increases in arterial stiffness and blood pressure following high-intensity resistance exercise. During the rest period, mid luteal phase trials exhibited significantly lower ftPWV, API, SBP and PP than early follicular phase trials, suggesting a phase-specific effect on vascular health. This observation is consistent with the well-documented vascular benefits associated with higher estrogen levels during the mid luteal phase, further supporting the phase-specific impact of hormonal fluctuations on vascular health.

Our study found that two hours after consuming dark chocolate, NO levels were significantly higher in the dark chocolate trial compared to the milk chocolate trial, while arterial stiffness and blood pressure were significantly decreased. Cocoa flavanols and epicatechins in dark chocolate were key contributors to these cardiovascular benefits, prior systematic reviews demonstrated that effective doses of cocoa flavanols (≥ 900 mg) and epicatechin (≥ 100 mg) lowered blood pressure by approximately 1–5 mmHg in normotensive individuals [35]. These effects were attributed to the stimulation of NO production, which enhanced vasodilation and endothelial function, ultimately leading to blood pressure reductions [36]. In our study, participants consumed an average of approximately 2424 mg of cocoa flavanols and 208 mg of epicatechin. These amounts substantially exceeded the thresholds reported in systematic reviews. Consistent with these findings, our study found that ftPWV, API, SBP, and PP significantly decreased two hours after dark chocolate consumption in both menstrual cycle phases. Additionally, the EF-MC trial exhibited significantly higher ftPWV, API, and SBP compared to EF-DC, ML-MC, and ML-DC trials, indicating that both estrogen and cocoa flavanols had protective effects. Notably, in the luteal phase, API and SBP in the ML-DC trial were significantly lower than in the ML-MC trial, further supporting the potential synergistic effect between estrogen and cocoa flavanols.

Furthermore, prior research indicates that consuming high- or low-cocoa-content beverages before moderate-intensity aerobic exercise can enhance flow-mediated dilation (FMD) and mitigate exercise-induced increases in blood pressure [37]. Previous research has also shown that 85% dark chocolate can attenuate blood pressure increases induced by acute stress in healthy adult women [17]. Cocoa flavanols also mitigated exercise-induced acute increases in blood pressure, likely through their ability to enhance NO production and improve endothelial function [38]. While these effects were primarily documented in the context of aerobic exercise, research on the effects of Cocoa flavanols supplementation following high-intensity resistance exercise remained limited. Consistent with these findings, we observed that dark chocolate consumption reduced vascular pressure following resistance exercise, particularly during the early follicular phase, where post-exercise ftPWV and API were significantly lower in the dark chocolate trial compared to the milk chocolate trial. However, no significant differences in arterial stiffness were observed immediately post-exercise (T0) during the mid-luteal phase. Notably, despite the synergistic effect between estrogen and cocoa flavanols observed at rest, this effect was not evident at T0 post-exercise. A possible explanation is the activation of the exercise pressor reflex (EPR), a mechanism that regulates cardiovascular responses during exercise by increasing SNS activity to maintain blood flow and perfusion pressure [39]. The heightened SNS activation induced by EPR elevates vascular resistance and may counteract the vasodilatory effects of estrogen or cocoa flavanols, thereby attenuating their synergistic impact on arterial stiffness post-exercise.

Our study revealed significant variations in arterial stiffness and blood pressure responses to high-intensity resistance exercise across menstrual phases. Specifically, the mid luteal phase trials exhibited a smaller increase in arterial stiffness and blood pressure post-exercise compared to the early follicular phase trials. Estrogen has been shown to enhance NO secretion and inhibit SNS activity, thereby improving endothelial function and mitigating arterial stiffness. These effects were particularly pronounced during the mid luteal phase when estrogen levels were at their peak [7, 9, 40]. Previous studies have demonstrated that high-intensity resistance exercise in women leads to significant increases in SBP and PWV within five minutes post-exercise [41]. This rise in arterial stiffness was attributed to the acute hypertensive response observed during high-intensity resistance exercise, which was associated with a significant reduction in arterial compliance [41]. Additionally, high-intensity resistance training also stimulates SNS activity, further promoting vasoconstriction and increasing arterial stiffness [42, 43]. Studies accounting for the menstrual cycle have reported significant increases in PWV at 30 and 60 min post-exercise during the follicular phase, with no such changes observed during the luteal phase [12]. PWV at 30 and 60 min post-exercise was significantly lower during the luteal phase compared to the follicular phase, indicating that women in the luteal phase are better able to mitigate exercise-induced increases in arterial stiffness [12]. These findings were consistent with our results, which showed that in the trials not consuming dark chocolate, SBP, ftPWV, and API significantly increased immediately post-exercise (T0). However, at the same time point, ftPWV and API were significantly lower during the mid luteal phase compared to the early follicular phase. This trend continued at T60, where ftPWV was again significantly lower in the mid luteal phase. These observations underscored the protective role of estrogen during the mid luteal phase in mitigating exercise-induced increases in arterial stiffness and blood pressure.

In our study, a significant increase in ftPWV, AVI and HR were observed in the EF-MC trial from rest to baseline. This phenomenon may be attributed to physiological changes associated with morning variations. Previous research has demonstrated that baPWV increases significantly from morning to midday, reflecting a circadian pattern in arterial stiffness [44]. Similarly, heart rate is influenced by diurnal rhythms, with a gradual increase during the day and a decrease at night [45]. These natural physiological fluctuations might have contributed to arterial stiffness and heart rate increased significantly from rest to baseline in the EF-MC trial, particularly in the absence of protective effects from estrogen or dark chocolate supplementation. Previous research has also demonstrated that central PWV significantly increases at 1, 10, 20, and 30 min post-exercise during both menstrual phases in healthy adult women [13]. These results align with our findings, where central arterial stiffness, represented by AVI, significantly increased at T0 and T60 across all trials.

Additionally, post-exercise hypotension (PEH) is a well-documented phenomenon, occurring as a result of increased heart rate and cardiac output during exercise, which enhances circulation efficiency and induces vasodilation, reducing vascular resistance [46]. Meta-analyses have shown that various exercise modalities, including aerobic, isotonic resistance, isometric resistance, and combined aerobic-resistance exercise, reduce blood pressure, with greater effects observed in hypertensive or prehypertensive individuals [6]. Our study observed a significant reduction in SBP at T60 and T120 post-exercise in the early follicular phase trials. Specifically, in the EF-MC trial, SBP decreased by 4 mmHg from REST to T60 and by 4.32 mmHg from REST to T120, while in the EF-DC trial, SBP decreased by 4.03 mmHg and 5.06 mmHg, respectively. These reductions were likely due to the higher resting SBP observed during this phase, which amplified the post-exercise hypotensive response. Furthermore, at T0, despite the absence of a synergistic effect on arterial stiffness, a significant reduction in SBP was observed in the ML-DC trial compared to the EF-MC trial, demonstrating a synergistic effect between estrogen and cocoa flavanols. This indicated that the regulatory mechanisms controlling arterial stiffness and blood pressure might have differed immediately post-exercise. Given the complexity of the EPR, which is a multifaceted mechanism regulating cardiovascular responses during exercise [39], further research is needed to elucidate its role in modulating post-exercise hemodynamic responses.

Furthermore, our findings highlighted significant differences in estrogen levels and their impact on cardiovascular markers, including NO, between the early follicular and mid-luteal phases. Higher estrogen levels in the mid-luteal phase were associated with lower resting values of ftPWV, API, and SBP. Prior research reported estrogen levels around 40 pg/mL in the early follicular phase and 120 pg/mL in the mid-luteal phase [47], which aligned with our findings of 66.53 ± 21.24 pg/mL (EF-MC) and 62.19 ± 24.06 pg/mL (EF-DC) in the early follicular phase, and 128.79 ± 58.54 pg/mL (ML-MC) and 127.11 ± 64.26 pg/mL (ML-DC) in the mid-luteal phase. This alignment supported the validity of the selected phases for assessing cardiovascular responses. Prior research has also suggested that higher estrogen levels stimulate NO production, leading to vasodilation, increased vascular elasticity, suppression of SNS activity, and reduced ET-1 production [7–9]. However, in the trials without dark chocolate supplementation, we did not observe significant differences in NO levels between the early follicular and mid-luteal phases. This suggests that estrogen may not substantially influence NO levels under the conditions of our study. Instead, the observed differences in arterial stiffness and blood pressure between menstrual phases may be attributed to other estrogen-mediated mechanisms, such as suppression of SNS activity and reduced ET-1 production, rather than enhanced NO production. Regarding PWV, previous studies have produced inconsistent results when examining PWV across different menstrual phases. Some studies found no differences in rest PWV values among different phases [10, 13]. However, other research has shown that baseline PWV values during the luteal phase (18–24 days) are significantly lower than those in the follicular phase (2–5 days) [10]. These discrepancies may arise from differences in how menstrual phases are defined. For instance, Augustine et al. [12] defined menstrual cycle phases as early follicular (1–7 days) and early luteal (14–19 days), while Adkisson et al. [9] used early follicular (2–4 days), late follicular (12–14 days), early luteal (17–19 days), and late luteal (25–28 days), none of which found significant differences in PWV. It is also important to note that estrogen levels not only increase before ovulation but also rise during the mid-luteal phase [48]. As a result, arterial stiffness measurements taken on different days of the same menstrual cycle may yield varying results, underscoring the importance of considering the duration and timing of the menstrual cycle in cardiovascular research.

### Limitations

This study did not confirm ovulation through direct methods, such as urinary luteinizing hormone (LH), progesterone testing or basal body temperature (BBT) monitoring. While phase identification relied on menstrual cycle length calculations, the absence of direct ovulation confirmation may have introduced variability in hormonal status. Additionally, blood samples were collected after the consumption of both the standard breakfast and chocolate supplementation, without measuring NO levels at complete rest. Although this design allowed for the assessment of changes in NO levels by comparing the milk chocolate and dark chocolate trials, it limited the ability to establish absolute baseline values. Moreover, the duration of eccentric and concentric phases during resistance exercises was not explicitly controlled. Participants were instructed to perform the movements in a controlled and deliberate manner under the supervision of a certified instructor to ensure safety and proper technique. Blood pressure was measured in a seated position, while ftPWV was assessed in a supine position. The transition between positions may have introduced minor variability, but efforts were made to minimize this impact. Furthermore, this study exclusively measured NO levels as the primary biomarker of vascular function. While NO plays a key role in endothelial function, the assessment of additional biomarkers such as ET-1, noradrenaline, and oxidative stress markers would have provided a more comprehensive evaluation of the interaction between estrogen and cocoa flavanols. Given the complexity of vascular regulation, future studies should incorporate multiple physiological markers and hemodynamic measurements at each time point to better elucidate the potential synergistic effects of estrogen and cocoa flavanols on vascular function and autonomic regulation.

## Conclusions

This study demonstrated that 85% dark chocolate supplementation improved vascular function in healthy adult women, with effects varying by menstrual phase and exercise conditions. At baseline, dark chocolate significantly reduced SBP, PP, ftPWV and API in both the early follicular and mid-luteal phases, likely due to its impact on NO production and enhanced endothelial function. Without dark chocolate supplementation, no significant differences in NO levels or vascular parameters were observed between the early follicular and mid-luteal phases. Post-exercise, dark chocolate attenuated immediate post-exercise increases in ftPWV and API during the early follicular phase, where estrogen levels were lower and hormonal support for vascular function was reduced. However, its effects were less pronounced during the mid-luteal phase, likely because higher estrogen levels already supported vascular function. These findings suggested that the benefits of cocoa polyphenols could have contributed to greater vascular benefits under conditions of reduced hormonal influence, warranting further research into their long-term effects and broader applications in cardiovascular health.

## Data Availability

The datasets used and/or analyzed during the current study are available from the corresponding author on reasonable request.

## Abbreviations

WHO: World Health Organization
CVD: Cardiovascular disease
NO: Nitric oxide
NOS: Nitric oxide synthase
ET-1: Endothelin-1
SNS: Sympathetic nervous system
L-NAME: L-nitroarginine methyl ester
1RM: Maximum repetition
ftPWV: Finger-toe pulse wave velocity
SBP: Systolic blood pressure
DBP: Diastolic blood pressure
API: Arterial pressure volume index
AVI: Arterial velocity pulse index
PEH: Post-exercise hypotension
EF-MC: Early follicular milk chocolate trial
EF-DC: Early follicular dark chocolate trial
ML-MC: Mid luteal milk chocolate trial
ML-DC: Mid luteal dark chocolate trial
